# Clinical Effects of Home Telemonitoring in the Context of Diabetes, Asthma, Heart Failure and Hypertension: A Systematic Review

**DOI:** 10.2196/jmir.1357

**Published:** 2010-06-16

**Authors:** Guy Paré, Khalil Moqadem, Gilles Pineau, Carole St-Hilaire

**Affiliations:** ^2^Agence d’évaluation des technologies et des modes d’intervention en santéQuebec Department of HealthMontrealCanada; ^1^HEC MontrealMontrealCanada

**Keywords:** Home telemonitoring, information technology, chronic illnesses, clinical effects

## Abstract

**Background:**

Home telemonitoring figures among the various solutions that could help attenuate some of the problems associated with aging populations, rates of chronic illness, and shortages of health professionals.

**Objective:**

The primary aim of this study was to further our understanding of the clinical effects associated with home telemonitoring programs in the context of chronic diseases.

**Methods:**

We conducted a systematic review which covered studies published between January 1966 and December 2008. MEDLINE, The Cochrane Library, and the INAHTA (International Network of Agencies for Health Technology Assessment) database were consulted. Our inclusion criteria consisted of: (1) English language publications in peer-reviewed journals or conference proceedings and (2) studies involving patients with diabetes, asthma, heart failure, or hypertension, and presenting results on the clinical effects of home telemonitoring.

**Results:**

In all, 62 empirical studies were analyzed. The results from studies involving patients with diabetes indicated a trend toward patients with home telemonitoring achieving better glycemic control. In most trials in which patients with asthma were enrolled, results showed significant improvements in patients’ peak expiratory flows, significant reductions in the symptoms associated with this illness, and improvements in perceived quality of life. Virtually all studies involving patients with hypertension demonstrated the ability of home telemonitoring to reduce systolic and/or diastolic blood pressure. Lastly, due to the equivocal nature of current findings of home telemonitoring involving patients with heart failure, larger trials are still needed to confirm the clinical effects of this technology for these patients.

**Conclusions:**

Although home telemonitoring appears to be a promising approach to patient management, designers of future studies should consider ways to make this technology more effective as well as controlling possible mediating variables.

## Introduction

The health systems of many countries are facing serious challenges concerning current and expected demographic trends that may far exceed their financial resources. The United Nations has reported that the world’s population will continue to age, reaching 9 billion by 2050, and in the developed countries, the number of people over 60 years of age is expected to almost double, from 245 million in 2005 to 406 million in 2050 [1]. Closely tied to this phenomenon of aging populations are rising rates of chronic illnesses such as heart failure, hypertension, chronic respiratory diseases, and diabetes, some of the factors that are driving the need to review how care is organized and the need to propose new interventions [2]. It is generally recognized that the chronically ill use medical, hospital, and emergency services more often. For instance, the Health Council of Canada has estimated that chronic illnesses are associated with 70% of Canadian hospital stays [3]. Furthermore, a shortage of health professionals has become a problem around the world, which also imposes certain constraints in almost all countries, rich and poor alike. According to World Health Organization estimates, 57 countries are experiencing acute shortages of health professionals [4]. The phenomenon of fewer health professionals suggests that services need to be restricted and priorities need to be set.

Information and communication technologies figure among the solutions that could help attenuate some of the problems associated with aging populations, rates of chronic illness, and shortages of health professionals, and, at the same time, facilitate service reorganization [5]. Greater use of telemedicine, for example, could make it easier to serve remote populations and lessen the impact of the lack of health professionals [6].

Home telemonitoring, the focus of this study, is an application of telemedicine in which physiological and biological data are transferred from the patients’ home to the telemonitoring center to monitor patients, interpret the data, and make clinical decisions [7]. Home telemonitoring is a relatively recent approach with growing numbers of applications, not only in many industrialized countries, but also in some developing countries [8]. The underlying goal is to organize this “telecare” approach according to case and care management principles and to substitute home telemonitoring for the integrated and continuous monitoring classically used to monitor patients during an episode of care. In many health care systems around the world, home telemonitoring is an integral part of a broader view of deinstitutionalization and reflects a societal orientation toward maintaining patients in their homes [9].

Paré et al [8] conducted a systematic review of the magnitude of several outcomes associated with home telemonitoring of patients with diabetes, pulmonary conditions (asthma, chronic obstructive pulmonary disease [COPD], and pulmonary transplantation), hypertension, and heart failure. That review provided evidence that confirmed the reliability and accuracy associated with home telemonitoring as an approach to patient management among patients with these diseases. In general, very few errors and technical problems were faced in the projects considered in the review. The earlier systematic review also presented consistent findings related to the effects of home telemonitoring on patients’ attitudes and behaviors. It appeared that most patients complied with telemonitoring programs because this approach allowed them to actively participate in the process of care, improved their feelings of security, and led to their empowerment. Of utmost importance, the authors reported some evidence of the positive effects of home telemonitoring on the patients’ conditions, especially in the cases of pulmonary conditions and heart failure. Given the importance of improving the medical condition of patients and their well-being with any approach to care, the authors recommended that the goal of future research be to evaluate the clinical effects of home telemonitoring.

In this regard, the primary objective of the present study was to update the systematic review conducted by Paré et al [8] and, most importantly, provide a deeper analysis of the clinical effects associated with home telemonitoring programs. We decided to focus on four chronic conditions: diabetes, asthma, heart failure, and hypertension, given the availability of empirical evidence on these illnesses. The second objective was to identify and discuss the main conditions for success when implementing home telemonitoring programs.

## Methods

Our systematic review, which followed the PRISMA guidelines [10], covered studies published from January 1966 through December 2008. The following three databases were consulted: MEDLINE (PubMed interface), The Cochrane Library, and the International Network of Agencies for Health Technology Assessment (INAHTA) database. For the purpose of this study, the inclusion criteria consisted of: (1) English language publications in peer-reviewed journals or conference proceedings, and (2) studies that presented results in terms of clinical effects and involved patients with diabetes, asthma, heart failure, or hypertension.

**Figure 1:**
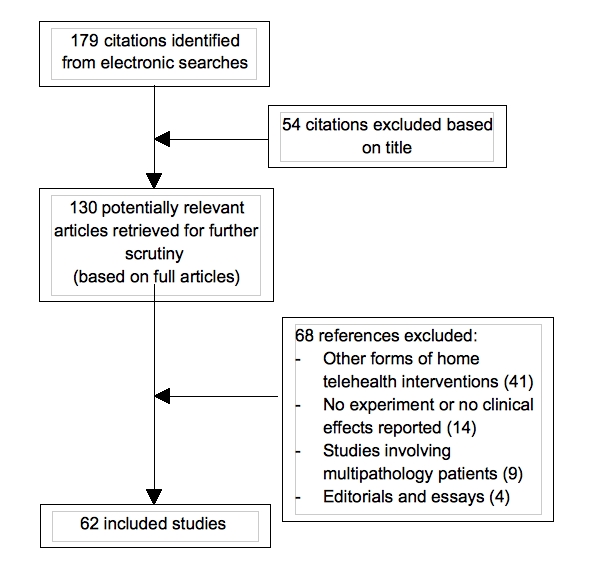
Selection of studies

We conducted the search using four keywords (home telemonitoring, home telecare, telehealth, and telehomecare) in conjunction with each of the following terms: diabetes, asthma, heart failure, and hypertension. The original search resulted in 179 articles after eliminating duplicate studies, systematic reviews, and meta-analyses. From these, 54 articles were deemed not relevant based on title. The remaining 130 articles were reviewed by 2 of the investigators to determine whether they were appropriate for inclusion. In this process, the reviewers relied on the following exclusion criteria: (1) other forms of home telehealth interventions (eg, studies that involved downloading of data during clinic visits or at the end of the study period, studies that included regular telephone calls by care providers, and studies that only considered teleconsultation delivered via video visits); (2) studies that did not involve home telemonitoring experiments and, for instance, focused on a detailed description of the technology deployed; (3) studies examining multipathology groups of patients; and (4) editorials or general essays. Of the 130 articles, 68 were excluded based on these criteria (Multimedia Appendix 1 lists the excluded studies). As a result, the final number of articles included in this review was 62. Figure 1 presents the flow diagram detailing the process of study selection and the characteristics and key findings of the included studies are presented in Multimedia Appendix 2. Importantly, using the same search strategy, studies published after the cutoff date (ie, December 31, 2008) were identified and their findings have been taken into account as complementary material and, as such, are described only in the Discussion section.

To meet our main objective, the first author developed a coding scheme for the articles. The completeness and reliability of the coding table was tested by randomly selecting 8 studies (ie, 13% of the sample) and then having the first 2 authors independently code them. This resulted in a 92% rate of agreement. The differences were reconciled by consensus. Following this pretest, some minor adjustments were made to the coding scheme.

The studies were analyzed in the following order: those on diabetes (n = 24), asthma (n = 8), heart failure (n = 17), and hypertension (n = 13). The strength of evidence in each study was judged using the classification system drawn by Jovell and Navarro-Rubio [11] in which study design is specified as one of 9 levels in descending order of strength (see Table 1). Each level is further qualified by conditions of scientific rigor for the study. We separated the trials under each chronic disease into three groups and analyzed each group separately. The first group included studies that provided the best evidence, that is, they corresponded to level 2 of the classification system. The second group, corresponding to level 3, included RCTs conducted with small samples (100 or fewer subjects in each arm). Finally, the third group corresponded to levels 4 to 9, representing mainly nonrandomized trials, cohort studies, and descriptive studies.

**Table 1:** Classification of study design from Jovell and Navarro-Rubio [11]

Level	Type of Study Design
1	Meta-analyses of randomized controlled trials
2	Large-sample randomized controlled trials
3	Small-sample randomized controlled trials
4	Nonrandomized controlled prospective studies
5	Nonrandomized controlled retrospective trials
6	Cohort studies
7	Case-control studies
8	Non-controlled clinical series, descriptive studies, consensus methods
9	Anecdotes or case reports

## Results

The data in Table 2 provide a general profile of our sample of 62 studies. The data show that home telemonitoring programs have appeared quite recently. Even though the first study on this subject was published in 1987, most early projects to be described in the literature began to appear in the early 1990s. Studies published between 1991 and 1995 represent 6% of the total sample. The number of published studies then grew in the second half of the decade (1996 to 1999), representing 15% of the sample. The number then increased significantly: more than three quarters of the studies in the sample were published after 2000. The data also show that 45% of the studies were carried out in the United States, approximately a third were conducted in Europe, while 6% were conducted in Asia and in Canada. Finally, almost three quarters of the studies in our sample were RCTs, both small and large.

The following subsections present and illustrate the nature and scope of the clinical effects associated with home telemonitoring programs. As explained above, these effects will be discussed in the specific context of the chronic illnesses included in the present analysis: diabetes, asthma, heart failure, and hypertension. Given the higher level of evidence provided by large RCTs, we highlight these findings in our analysis.

**Table 2:** Profile of the sample

		Diabetes	Asthma	Heart Failure	Hypertension	Full Sample
		(n=24)	(n=8)	(n=17)	(n=13)	(n=62)
**Year of publication**						
	Prior to 1991	-	-	-	1	1 (2%)
	1991-1995	4	-	-	-	4 (6%)
	1996-2000	3	1	2	3	9 (15%)
	2001-2004	12	4	7	5	28 (45%)
	2005-2008	5	3	8	4	20 (32%)
**Where the study was conducted**						
	United States	11	3	8	6	28 (45%)
	Europe	10	2	6	3	21 (34%)
	Asia	2	1	-	1	4 (6%)
	Canada	1	-	2	1	4 (6%)
	Elsewhere	-	2	1	2	5 (8%)
**Type of publication**						
	Journal article	24	8	16	13	61 (98%)
	Proceeding	-	-	1	-	1 (2%)
**Research design**						
	Large RCT	2	1	5	3	11 (18%)
	Small RCT	19	5	8	2	34 (55%)
	Nonrandomized study	3	2	4	8	17 (27%)

### Diabetes

In the first of two large RCTs in our sample, patients were followed by two general medicine clinics in a single county in California [12]. Veterans with a diagnosis of diabetes mellitus or who had an active prescription for a hypoglycemic agent were identified. Excluded were patients who were more than 75 years of age and patients who had a diagnosed psychotic disorder, a disabling sensory impairment, or a life expectancy of less than 12 months. Patients were randomly assigned to usual care or to receive an intervention that consisted of a series of automated telephone calls designed to identify patients with health and self-care problems and to deliver targeted and tailored self-care education messages. The calls consisted of hierarchically structured messages composed of statements and queries recorded with a human voice. During each assessment, patients were asked to report information about self-monitored blood glucose readings, perceived glycemic control, symptoms of poor glycemic control, foot problems, chest pain, and breathing problems. At the end of each assessment, patients were given the options of listening to a randomly cycling diabetes “health tip” and of participating in an interactive self-care education module focused on diet and weight control. On a weekly basis, the automated system generated reports organized according to the urgency of reported problems, and a nurse used these reports to prioritize patient contacts. During follow-up calls, the nurse not only addressed problems reported during the assessments but also provided more general self-care education. Patients assigned to the usual care control group had no systematic monitoring between clinic visits and received no reminders of upcoming clinic appointments.

Clinical effects were collected at 12 months for 89% of the patients (n = 248). Patients in the intervention group (n = 124) reported better glycemic control (*P* = .01) and fewer diabetic symptoms (*P* < .001) at follow-up than patients in a control group that received usual care. While telemonitored patients had minimally lower HbA1c levels (0.3%, *P* = .1) at follow-up than patients in the control group, the proportion of patients with normal HbA1c levels in the intervention group increased by 9% (17% vs 8%, *P* = .04), while serum glucose levels among these patients decreased by 41 mg/dL compared with baseline (180 vs 221 mg/dL, *P* = .005). Based on these findings, Piette et al [12] concluded that automated calls represented an effective strategy for improving glycemic control and for controlling symptoms among vulnerable patients with diabetes.

In the second large RCT, Shea et al [13] conducted a study comparing home telemonitoring with usual care in 1665 Medicare recipients with diabetes aged 55 years or over and living in federally designated, medically underserved areas of New York State. Excluded were patients with moderate or severe cognitive, visual, or physical impairments or who had severe comorbid disease. Participants randomized to the intervention group (n = 844) received a home telemedicine unit (HTU). The HTU consisted of a Web-enabled computer with a modem connection to an existing telephone line. The HTU provided four major functions: (1) videoconferencing over plain old telephone service (POTS) connections, allowing patients to interact with nurse case managers; (2) remote monitoring of glucose and blood pressure; (3) dial-up Internet service provider access and secure Web-based messaging with nurse case managers; and (4) access to an educational website created for the project by the American Diabetes Association. Patients in the usual care group (n = 821) remained under the care of their primary care providers.

In the intervention group, the study found that, within one year, mean HbA1c had improved from 7.35% to 6.97%. In a subgroup with baseline HbA1c greater than or equal to 7%, HbA1c improved from 8.35% to 7.42% (n = 353). In the usual care group, within one year mean HbA1c had improved from 7.42% to 7.17%. Adjusted net reductions favoring the intervention were as follows: HbA1c, 0.18% (*P* = .01); systolic and diastolic blood pressure, 3.4 mmHg (*P* = .001) and 1.9 mmHg (*P* < .001); and LDL cholesterol, 9.5 mg/dL (*P* <. 001). Based on these results, the telehomecare program improved patients’ glycemic control, blood pressure levels, and total and LDL cholesterol levels after one year of follow-up.

A total of 19 small RCTs examined the effects of home telemonitoring programs on patient outcomes. As indicated in Multimedia Appendix 2, a significant decrease in HbA1c was observed in 10 of these studies for patients in the home telemonitoring groups [14-23]. For instance, Welch et al [20] reported that the mean HbA1c change score for the intervention group (n = 26) was statistically significant at 6 months (*P* = .001) and at 12 months (*P* <. 001), while the usual-care group (n = 26) showed small improvements that were not significant at either 6 or 12 months. These results indicated that the intervention, which focused on blood glucose control and insulin adjustment, was clinically useful in reducing HbA1c. As another example, Lavery et al [17] reported a significant decline in the number of diabetic foot complications in a group using hand-held infrared skin thermometers (*P* =. 01).

These findings were not consistent, however, with the results reported in 9 small RCTs that found that electronic transmission of blood glucose levels was equally as effective as standard or conventional outpatient management [24-32]. For instance, Chase et al [24] did not find significant differences in diabetes complications (eg, hypoglycemia) in their sample of adolescent diabetic patients. They observed that electronic transmission of blood glucose levels and other diabetic data every 2 weeks—in lieu of a clinic visit—resulted in a similar level of glucose control and incidence of acute diabetes complications when compared with current standard care. As another example, Ladyzynski and Wojcicki [32] observed less variability in glycemic control among the patients in the home telecare group. This indicated that the home telecare system helped patients to better comply with their physician’s recommendations to maintain glycemic control. Nevertheless, the mean level of metabolic control and the insulin dose adjustment patterns were very similar in both groups, regardless of a much higher reporting frequency of blood glucose levels in the intervention group.

Finally, three nonrandomized studies [33-35] also reported better glycemic control with home telemonitoring.

### Asthma

The data in Table 2 show that our sample included 8 studies associated with asthma, including one large RCT. The RCT was conducted by Rasmussen et al [36] with a sample of 300 Danish adults randomized to three groups: (1)home telemonitoring by Internet (the intervention group), (2)monitoring by a specialist, and (3)monitoring by a general practitioner. After 6 months, the authors found that fewer asthma symptoms were reported by patients in the intervention group than in either the group monitored by specialists (*P* = .002) or the group monitored by a general practitioner (*P* = .001). The intervention group also reported better quality of life compared with the groups monitored by the specialists and the general practitioners (*P* = .03 and *P*= .04, respectively) as well as better pulmonary function (*P* = .002 and *P* = 001, respectively). In summary, this large RCT suggested that a patient’s asthma was better controlled when physicians and patients used an interactive tool to monitor asthma over the Internet.

The positive results reported above were confirmed in 4 of the 5 small RCTs that included asthmatic patients [37-40]. For instance, Jan et al [37] assessed the effectiveness of an Internet-based interactive asthma educational and monitoring program. At the end of this 3-month trial, compared with conventional asthma management (n = 76), the Internet group (n = 88) had fewer nighttime symptoms (*P* = .03) and daytime symptoms (*P* = .01); improved peak expiratory flow (PEF) in the morning (*P* =. 02) and at night (*P* = .01); and improved quality of life (*P* = .05). In another example, Guendelman et al [38] observed that the odds of having any limitation in activity during the 90-day trial were significantly lower (*P* = .03) for children randomized to an Internet group (n = 66) than among children in a control group (n = 68). The intervention group was significantly less likely to experience PEF readings that indicated a severe asthma exacerbation or that indicated the child’s asthma was not under sufficient control and required additional medication (*P* = .01). Urgent calls to the hospital were also significantly less likely in the intervention group (*P* = .05).

Only one small RCT did not produce significant results. Indeed, Willems et al [41] found nonsignificant differences between the experimental group (n = 55) and the control group (n = 54) in terms of asthma symptoms and quality of life. According to the authors, there were two main reasons for these findings: infrequent data transmission (once a month) and the low to moderate severity of asthma among participants.

Lastly, positive and significant clinical outcomes associated with home telemonitoring were observed in one small nonrandomized trial [42] and in one cohort study [43].

### Heart Failure

Most studies concerned with home telemonitoring of heart failure patients have considered either patient outcomes (eg, mortality rates and quality of life) or quality metrics reflecting efficiency in care delivery (eg, hospital readmission rates, emergency room visits, and length of stay) or both. We will begin by highlighting the findings of large RCTs and then present the main trends found in the small RCTs and nonrandomized studies.

In the first large RCT, 280 patients from 16 heart failure centers across the United States were randomly assigned to the intervention group or to the control group. The 138 participants in the intervention group were provided with a home monitoring system and the 142 participants in the control group received standard care [44]. The home monitoring system included an electronic scale placed in the patient’s home and an individualized symptom response system, which was linked via a standard phone line. Patients were instructed to weigh themselves and to answer yes/no questions about heart-related symptoms twice daily. Over the course of the 6-month follow-up period, there were 26 (18.4%) deaths in the control group and 11 (8.0%) deaths in the intervention group, representing a 56.2% difference in mortality rates (*P* < .01). However, no significant difference was found between the two groups in terms of time to death or first readmission to hospital (*P* = .16). Further, patients in both groups experienced improvements between quality of life scores obtained at baseline and at the 6-month follow-up. Although none of the differences were statistically significant, the intervention group tended toward improvements in all the quality of life measures. Finally, no significant differences were observed between the intervention and control groups in terms of time to first emergency department visit, total number of emergency department visits, or total number of cardiovascular hospitalizations.

In a second study, conducted by Benatar et al [45], 216 patients with heart failure were randomized to one of two home health care delivery methods for 3 months after discharge from hospital. Care was delivered either through home nurse visits or a nurse telemanagement method. Patients in the nurse telemanagement group (the intervention group) used telephone-linked home monitoring devices to measure their weight, blood pressure, heart rate, and oxygen saturation level. These data were transferred daily to a secure Internet site. When a patient’s physiological data exceeded preestablished limits, an alarm would be automatically transmitted to an alphanumeric pager carried by an advanced practice nurse. The results of the study showed that quality of life as measured by the Minnesota Living with Heart Failure Questionnaire was significantly improved for both groups. However, the researchers observed a trend toward greater improvement in quality of life in the nurse telemanagement group compared with the control group. More specifically, the mean score on the quality of life questionnaire fell from 77.9 to 51.6 (lower scores indicate better quality of life) in the intervention group (*P* < .01) compared with a decrease from 77.2 to 57.7 in the control group (*P* < .01). Importantly, patients in the intervention group had fewer hospital readmissions for heart failure (*P* < .001) and shorter lengths of stay in hospital (*P* < .001) compared with the control group.

Third, Cleland et al [46] sought to identify whether patients allocated to a home telemonitoring group (the intervention group) would provide improved outcomes compared with nurse telephone support (control group 1) and usual care (control group 2) for patients with heart failure who were at high risk of hospitalization or death. Patients with a recent admission for heart failure and a left ventricular ejection fraction less than 40% were assigned randomly to the intervention group, control group 1, or control group 2 in a 2:2:1 ratio. The intervention group (n = 106) used automated devices to send self-measurements of weight, blood pressure, heart rate, and heart rhythm twice daily to a cardiac center. Control group 1 consisted of patients for whom specialist nurses were made available by telephone (n = 110). Control group 2 consisted of patients for whom primary care physicians delivered the usual care (n=55). During 8 months of follow-up, higher mortality was observed among the patients assigned to receive usual care than among the patients assigned to receive nurse telephone services or home telemonitoring (*P* = .03). In terms of service utilization measures, the number of readmissions was similar between patients in control group 1 and the telemonitoring group, but for readmitted patients, the mean length of stay was 6 days less for the group with home telemonitoring compared with mean length of stay for readmitted patients in control group 1 (no *P* value reported).

Fourth, the Heart Failure Home Care trial was a multicenter randomized controlled trial of enhanced versus routine heart failure monitoring in Medicare-eligible patients who were women and/or racial minorities [47]. Inclusion criteria included, but were not limited to, Medicare beneficiaries greater than 65 years of age who had been discharged from hospital with a primary or secondary diagnosis of heart failure within 6 months of randomization. A total of 315 patients were randomly assigned to two groups: 160 patients received a home monitoring system and the control group consisted of 155 patients who received standard care. Patients in the intervention group were asked to weigh themselves daily and respond to questions concerning heart failure symptoms. The monitored group transmitted their information to a telemonitoring center. When a patient’s weight exceeded a preestablished limit, a nurse would contact the patient and notify the attending physician. All participants were provided with educational materials and information as to when they should seek medication attention. The compliance rate associated with electronic data transmission of patients’ weights and symptoms of heart failure was very high at 97%. The incidence of the primary outcome, 6-month cardiac mortality, or readmission for heart failure, was not statistically different between the control and intervention groups (*P* = .15). Emergency room visits were common in both groups, and the number of emergency room visits was comparable across groups (*P* = .43). In short, this study was unable to find a benefit from home telemonitoring as compared with the traditional home care model over a 6-month period.

The fifth and last large RCT evaluating the health effects of home telemonitoring of patients with heart failure was conducted by Dansky et al [48] in 10 home care agencies in the same US state. The patient sample consisted of 2 experimental groups and a control group in each of the 10 agencies. The first group allocated to home telemonitoring (experimental group 1) consisted of patients who were each given a terminal to transmit daily their blood pressure, weight, and heart rate to their home care agency. The second home telemonitoring group (experimental group 2) consisted of patients who were given the same type of terminal as the first group in addition to a video camera, which was used 2 or 3 times a week for a remote consultation with a nurse. In all, 284 patients participated in the study as follows: 112 in the control group, 127 in experimental group 1 and 45 in experimental group 2. The outcomes of interest were control of the symptoms associated with heart failure and mortality. Over the 120-day follow-up period, the mortality rate was similar between the control group and experimental group 1 (*P* = .11) and between the control group and experimental group 2 (*P* = .47). However, the reduction in symptoms was more pronounced in the patients in experimental group 1 than in the other two groups, both for symptoms associated with diet (*P* = .04) and those associated with their use of medication (*P* = .001). There was also a tendency for patients in the home telemonitoring groups to have fewer hospitalizations at two points in time, at 60 and 120 days; however, the differences were statistically significant only at 60 days (*P* = .01). Lastly, patients in both home telemonitoring groups had fewer emergency room visits than patients in the control group. At 60 days, approximately 30% of the control group had had an emergency room visit, compared with 24% of experimental group 1 and 18% of experimental group 2 (*P* = .01). The differences were less striking at 120 days, but followed the same pattern.

We found 7 small RCTs and 3 nonrandomized studies in which *P* values were reported [49-58]. We observed that 9 of these studies measured the effects of home telemonitoring on patients’ quality of life or symptoms. All except 2 of these studies [53,56] found an improvement in quality of life or a reduction in symptoms over the course of the intervention in the patients followed by home telemonitoring. The *P* values presented in these studies varied from .002 to .05. However, the two studies in which mortality was the outcome of interest were unable to demonstrate a statistically significant difference in favor of the home telemonitoring group [52-53]. A small RCT [59] and a nonrandomized study [60] did not report *P* values.

In addition to the large RCTs, 3 small RCTs and 2 cohort studies examined the effects of home telemonitoring on health services utilization. All 3 small RCTs [51,52,55] reported no significant differences in the number of readmissions or length of stay between the telemonitoring intervention group and the control group receiving usual care On the other hand, the number of readmissions and the number of days of hospitalizations for chronic heart failure among the participants in both cohort studies [57-58] decreased significantly during the 12-month study period (*P* <. 001).

### Hypertension

We found 3 large RCTs that examined populations of patients with hypertension. In the first, Friedman et al [61] evaluated the effects of automated telephone patient monitoring and counselling on patient adherence to antihypertensive medications and on blood pressure control. The randomized trial was conducted in 29 communities in the greater Boston area. The study subjects were 267 patients recruited from community sites who were over 60 years of age, on antihypertensive medication, had a systolic blood pressure (SBP) greater than 160 mmHg and/or a diastolic blood pressure (DBP) greater than 90 mmHg. Patients were excluded if they had a life-threatening illness, did not speak English, did not have a telephone, or were unable to use a telephone. The study compared subjects who received usual medical care (n = 134) with those who used a computer-controlled telephone system in addition to their usual medical care (n = 133) over a period of 6 months. Each week, subjects in the intervention group reported self-measured blood pressures, knowledge of and adherence to antihypertensive medication regimens, and medication side-effects. This information was sent to their physicians. Results indicated that mean antihypertensive medication adherence improved 17.7% in the intervention group and 11.7% in the control group (*P* = .03). Furthermore, mean DBP decreased 5.2 mmHg in the intervention group compared with a mean decrease of 0.8 mmHg in the control group (*P* = .02). Among the intervention group, mean DBP fell more among participants who had improved adherence to their medication regime (*P* = .03).

In the second RCT, Artinian et al [62] tested the hypothesis that individuals who participated in usual care plus blood pressure (BP) telemonitoring (the intervention group) would have a greater reduction in BP from baseline to 12-month follow-up than would individuals who received usual care only (the control group). A two-group, experimental, longitudinal design was used with block-stratified randomization. African Americans with hypertension were recruited through free BP screenings offered in the community. Data were collected at baseline and at 3-, 6-, and 12-month follow-ups. Results indicated that the intervention group (n = 167) had a greater reduction in SBP (13.0 mmHg) than the control group (7.5 mmHg; *P* = .04) from the baseline to the 12-month follow-up. Although the reduction in DBP was greater in the intervention group (6.3 mmHg) compared with the control group (4.1 mmHg), the difference was not statistically significant (*P* = .12).

The third RCT was conducted by Madsen et al [63]. Hypertensive patients recruited by general practitioners participated in the study. Blood pressure of participants in the intervention group (n = 105) was telemonitored from patients’ homes. In the control group (n = 118), patients received usual care with office visits to monitor blood pressure. After 6 months, participants filled out the Short-Form-36 Health Survey to assess quality of life. Patients in the telemonitoring group had higher mean scores in the bodily pain domain than patients in the control group, indicating less pain and interference with activities among telemonitored patients (*P* = .03). In both groups, systolic BP decreased significantly from baseline to follow-up. The decrease was -11.9 mmHg in the intervention group and -9.6 mmHg in the control group (mean difference of -2.3, *P* =.23). As a result, the authors concluded that antihypertensive treatment based on telemonitoring of home BP was as effective at reducing BP as usual office BP monitoring.

The two small RCTs in our sample [64-65] confirmed the positive outcomes of home telemonitoring in hypertensive patients. For example, in the study by Rogers et al [63], the intervention group consisting of 60 patients, and the control group consisted of 61 patients. The results indicated that blood pressure fell 2.8 mmHg among the telemonitored patients and rose 1.3 mmHg among usual care patients (*P* = .01 for the difference between the groups). The mean diastolic BP fell 2.0 mmHg in the experimental group but rose 2.1 mmHg among patients in the control group (*P* = .01 for the difference between the groups). Furthermore, mean systolic BP fell 4.9 mmHg in the group with home telemonitoring versus 0.1 mmHg in the group with usual care (*P* = .05).

Finally, 8 nonrandomized studies [66-73] also evaluated the clinical effects of home telemonitoring in hypertensive patients, of which 7 reported *P* values. The results of all of these studies indicated that home telemonitoring appeared to have benefits as shown by the clinical effects that were measured.

## Discussion

This section summarizes and discusses our main findings. First, the results from the 24 diabetes studies indicated a trend towards better glycemic control. Positive outcomes were observed in both large RCTs as well as in 13 other studies, including 10 small RCT studies. There were 9 other studies that concluded that home telemonitoring is as effective in glycemic control as the traditional approach to home follow-up. Overall, our findings are consistent with recent systematic reviews and meta-analyses on home telemonitoring for diabetes management, for example, the reviews by Paré et al [8] and Polinesa et al [74]. As shown in Multimedia Appendix 2, most studies included in the present review included patients with insulin-dependent diabetes mellitus (IDDM) and, hence, results might not be generalizable to other types of diabetes. In addition, it was not clear from the results of these 24 studies whether improvement in the clinical condition of patients was the result of the use of the technology itself or because of other factors. For instance, the positive outcomes observed in the study by Shea et al [13] might also be associated with the intensified provider consultation and/or the increased access to educational material. Similarly, Stone et al [75] found that active medication management by a nurse practitioner along with home telemonitoring demonstrated reductions in HbA1c after 3 and 6 months. Future research should therefore assess the relative impact of other potentially mediating variables or conditions on the clinical outcomes observed.

Second, as for asthma, 5 of the 6 RCTs included in this systematic review showed a significant improvement in PEF, a significant reduction in the symptoms associated with this illness, and a large improvement in perceived quality of life. Overall, our findings are aligned with a recent systematic review of home telemonitoring and respiratory conditions [76]. While these results may be encouraging, it is unclear whether the use of technology either promotes the resolution of symptoms, empowers the patient to self-manage their condition, or both. We concur with Smith et al [77] that studies are needed that address how the use of patient monitoring technology leads to self-management.

Third, home telemonitoring also provided for better control of blood pressure than the traditional home follow-up model. The findings from 4 of the 5 RCTs and 7 of the 8 nonrandomized studies of strategies to control blood pressure suggested that home telemonitoring does a better job of improving state of health in hypertension patients than other approaches. It is worth noting that in most cases the studies found a significant drop in blood pressure in the first 3 months of remote monitoring. While our findings are consistent with those reported elsewhere, for example the reviews by Paré et al [8] and Jaana et al [78], very few studies have presented changes in compliance with medication regimens and quality of life associated with home telemonitoring. A recent trial conducted by Parati et al [79] also confirmed the positive outcomes observed in this review. In that study, 329 hypertensive patients were randomized to either usual care on the basis of office blood pressure (the control group, n = 113) or to integrated care on the basis of teletransmitted home blood pressure (n = 216) and were observed over a period of 6 months. Results indicated that the percentage of daytime blood pressure readings that were within the normal range during the study period was higher in the group that teletransmitted their blood pressure readings than in the control group (*P* < .05). However, no significant between-group differences were found in the rate of change in treatment regimens prescribed by the physicians. Quality of life also tended to be higher in the intervention group, but the difference was not statistically significant. As a final remark concerning studies of home monitoring of patients with hypertension, the positive outcomes observed must be interpreted with caution because most trials were nonrandomized and several studies had small sample sizes.

The positive effects reported for diabetes, asthma, and hypertension are mainly associated with the fact that, by its very nature, telemonitoring allows for more frequent follow-up of patients and, as a result, may provide earlier detection of warning signs that a patient’s state of health is deteriorating [8]. However, many studies of heart failure have failed to show a reduction in either mortality or hospitalization rates, although a few trials have reported a trend towards shorter lengths of stay in hospital, for example, the studies of Benatar et al [45] and Cleland et al [46]. These findings are consistent with those reported by Paré et al [8] as well as the findings of two recent RCTs. In the Home-HF study [80], 182 patients with a recent hospitalization for heart failure were randomly assigned to daily telemonitoring or to a control group that received a package of intensive, conventional expert care. Although the study did not find significant differences between the two groups in survival (number of days) or in the number of days out of hospital, the results confirmed that home telemonitoring allowed early detection of worsening symptoms (*P* < .01). Similar to previous RCTs, for example the studies by Goldberg et al [44] and Benatar et al [45], the study failed to show an impact on quality of life. In another recent study, the Home or Hospital in Heart Failure trial [81], patients with a hospitalization for heart failure in the previous year were randomly assigned either to usual care (n = 160) or to home telemonitoring (n = 301). Mortality and length of stay were low in both groups and did not differ significantly.

### Critical Success Factors

Given the state of knowledge in this area, it becomes pertinent and important to examine the main conditions for a successful home telemonitoring program. These conditions are related to: (1) the patients targeted by the telemonitoring intervention, (2) the technological devices used, and (3) the characteristics of the telemonitoring program and work organization. Meeting the conditions described below may increase the likelihood of positive and statistically significant clinical effects.

First, with respect to the patients targeted by home telemonitoring programs, it needs to be determined whether home telemonitoring is suitable to everyone. On the basis of the studies in our sample, this would not appear to be the case. Several exclusion criteria were used in these studies. Patients were often excluded if they had a moderate or serious cognitive, visual, or physical disability. Also commonly excluded were patients who did not own a phone or who had a life expectancy measured in months rather than years. When determining eligibility criteria, it cannot be denied that some patients appear to benefit more than others. Several studies have suggested that the beneficial effects on state of health are observed mostly among patients whose state of health is considered serious (eg, the studies by Kwon et al [23] and Trappenberg et al [82]); patients who want to play an active role in the management of their illness (eg, the studies by Madsen et al [63], Rickerby and Woodward [83], DelliFraine and Dansky [84], and Hopp et al [85]); and patients who are interested in using this type of technological device (eg, the studies by Vähätalo et al [27], and Madsen et al [63]).

In terms of the technology, the user-friendliness of the device installed in the home and its nonintrusiveness in the lives of patients, particularly for the youngest patients, appear to be important acceptance criteria. Given the fact that the patients with chronic disease who are targeted in home telemonitoring applications do not all have the same level of technological skill, the same level of education, the same professional constraints, or the same lifestyle, and that some may have a slight visual or motor deficit, it would be preferable for application providers to ensure that patients have the technological device best suited to their specific needs. For some, a secure Web link will represent the best solution, whereas for others a cellular phone will be the most appropriate technology. Furthermore, the use of electronic measurement instruments is becoming increasingly common. Such instruments not only simplify data entry and transfer, they also provide more reliable data. As suggested by Dansky et al [48], empirical studies comparing various technologies (eg, Internet-based versus telephone-based) would provide important information for the advancement of chronic illness management.

Finally, certain issues appear to be associated with the tension that is created when telehomecare is added to home care services. The authors of a few studies (eg, studies by Gomez et al [16], Montori et al [21], and Biermann et al [29]) have suggested that the implementation of a telehomecare program requires a review of work organization to ensure a quick response to an alert from the technology as well as of a review of work organization planned around standard interventions. It is therefore important to plan for and then assign one or more nursing resources (depending on the number of patients followed) to monitor the clinical data received every day and take the required actions, as, for example, in the studies by Ahring et al [13] and Knox et al [86]. Moreover, a home telemonitoring application must be designed and implemented with the understanding that it is a complementary intervention and not a solution that replaces primary care [12]. Furthermore, telemonitoring completes and consolidates the health care system by allowing a continuum of care based on patient needs. Many of the telemonitoring programs that produced conclusive clinical results maintained their patient follow-up by telephone or in the hospital, as, for example in the studies by Shae et al [12], Shultz et al [17], and Jan et al [36]. Periodic visits to a medical clinic and home visits by nurses are also maintained, but their frequency may be adjusted based on changes in a patient’s state of health. The idea is that the technological device is not a substitute for follow-up of chronically ill patients by a health professional, rather such devices are used as leverage to improve the effectiveness and quality of professionals’ work.

### Limitations

Despite our use of a thorough search strategy, some empirical studies on home telemonitoring interventions may not have been identified for this review. Specifically, we did not examine the gray literature (unpublished documents and reports) on this topic; we focused instead on data that had been published through the peer-review process. Importantly, a meta-analysis was not possible due to the various data collection methods and outcomes in the reported studies. As well, it was not clear throughout the studies examined herein whether improvement in the clinical condition of patients was the result of the use of the technology itself or of other mechanisms, such as the intensified provider consultation or the greater access to education material. Future research should assess the impact of other potentially mediating variables or conditions on the clinical outcomes observed.

In spite of these limitations, this is the first systematic review to our knowledge that specifically examines the clinical outcomes of home telemonitoring programs across a variety of chronic conditions and addresses the critical success factors associated with such interventions. Insights regarding clinical outcomes of this emerging intervention and possible ways of making it more effective are presented in an organized manner, and future research directions in this area are recommended based on this systematic review.

### Conclusion

In the interests of providing appropriate support to the growing offer of home care services for the chronically ill and to maximize the associated benefits, health care organizations and professionals must, in our opinion, incorporate information technologies. Home telemonitoring, which requires the active participation of patients, constitutes a case in point. This mode of intervention allows for closer monitoring of each patient’s condition, as well as early detection of warning signs that a patient’s health is deteriorating. The findings of empirical studies conducted so far are encouraging. The results of a large majority of studies indicated better glycemic control and improved control of asthma and blood pressure. However, due to the equivocal nature of current findings pertaining to the clinical effects of home telemonitoring in the context of heart failure, larger trials are needed to confirm the benefits of this technology for these patients.

## Multimedia Appendix 1

List of excluded studies

## Multimedia Appendix 2

List of the 62 included studies

## Abbreviations

BP: blood pressure
COPD: chronic obstructive pulmonary disease
DBP: diastolic blood pressure
HbA1c: hemoglobin A1c
HTU: home telemedicine unit
IDDM: insulin-dependent diabetes mellitus
INAHTA: International Network of Agencies for Health Technology Assessment
PEF: peak expiratory flow
POTS: plain old telephone service
SBP: systolic blood pressure
