# Supplementary material for: Clinical Effects of Home Telemonitoring in the Context of Diabetes, Asthma, Heart Failure and Hypertension: A Systematic Review
Source: J Med Internet Res. 2010 Jun 16;12(2):e21. doi: 10.2196/jmir.1357 (PMC2956232; doi:10.2196/jmir.1357)
Supplement: Supplementary file 1 [file jmir_v12i2e21_app1.pdf]

Multimedia Appendix 2: List of 62 included studies

**DIABETES**	
**Large RCTs with support**	
Piette et al. [9]	HbA1c levels were 0.3% lower in the intervention group (p=0.1), and about twice as many intervention patients had HbA1c levels within the normal range (p=.04). Intervention patients reported better glycemic control (p=0.005) and fewer diabetic symptoms (p<0.0001) than control group patients.
Shea et al. [10]	Adjusted net reductions favoring the intervention group were as follows: HgbA1c, 0.18% (p=0.006); systolic and diastolic blood pressure, 3.4 (p=0.001) and 1.9 mm Hg (p<0.001); and LDL cholesterol, 9.5 mg/dL (p<0.001).
**Small RCTs with support**	
Ahring et al. [11]	In the experimental group, HbA1c significantly improved after 6 weeks (0.094 ± 0.012 vs 0.106 ± 0.028, p=0.05). After 12 weeks in the study, the HbA1c (0.092 ± 0.0011) was further and significantly (p=0.05) reduced compared with week 0. The control group showed a nonsignificant (p=0.10) improvement in HbA1c, after both 6 and 12 weeks compared with the start of the study.
Billiard et al. [12]	HbA1c declined significantly (p=0.05) during the telematic period compared with the booklet period. Such declined was not counterbalanced by weight gain or increased insulin dosages.
Gomez et al. [13]	The median value of HbA1c was 8.1 (6.6 - 9.1) at the beginning of the control study and 8.4 (6.9 – 9.1) at the beginning of the DIABTel study. The HbA1c was 8.15 (5.9 – 9.9) at the end of the control study and 7.9 (6.6 – 8.9) at the end of the DIABTel study. These findings show a trend towards reduction of the HbA1c after the DIABTel study (p=0.05).
Lavery et al. [14]	The experimental group had significantly (p=0.01) fewer diabetic foot complications (ulcers and Charcot fractures).
Shultz et al. [15]	For the intervention group, the gHbA1c dropped significantly (p<0.003) at 15 months whereas no change overall was found in the control group.
Tsang et al. [16]	A significant improvement in glycemic control was achieved during intervention compared with control periods (mean HbA1c reduction of 0.825%, p<0.019).
Welch et al. [17]	HbA1c change score for the intervention group was statistically significant at 6 months (-0.37% ± 0.57%, p<0.002) and at 12 months (-0.43% ± 0.59%, p<0.001). The control group had small improvements that were not significant at both 6 and 12 months.

Montori et al. [18]	Compared with the control group, telecare patients had a significantly lower 6-month HbA1c (8.2 vs. 7.8%, p=0.03) after accounting for HbA1c at baseline.
Wojcicki et al. [19]	Better glycemic control in the study group in comparison with the control group during the course of treatment, as assessed by the average difference of the mean blood glucose level (MBG) and J indices calculated weekly (Δ MBG = -3.2 ± 4.3 mg/dL, p=0.0016; Δ J indices = -1.4 ± 2.3, p=0.0065).
Kwon et al. [20]	HbA1c levels were significantly decreased from 7.59 to 6.94% with the intervention group (p<0.001). At the end of the study, HbA1c levels in the intervention group were significantly lower than in the control group after adjusting the baseline HbA1c (p<0.001).
**Small RCTs with no support**	
Chase et al. [21]	HbA1c values significantly decreased in both groups (control and intervention), with no statistically significant difference between groups (p=0.96).
Chumbler et al. [22]	No significant differences were observed between the 2 groups in any of the clinical outcomes measures (HbA1c, insulin use, systolic and diastolic blood pressure).
Marrerro et al. [23]	There was no significant between-group difference in metabolic control, although levels in both groups increased over time (p=0.001). According to the authors, the non-significant results are due to hormonal changes and puberty.
Vahatalo et al. [24]	There was a slight overall increase in the HbA1c during the study which was similar to both groups. According to the authors, the reason for this non significant result is probably mainly the low measurement activity of most of the intervention patients.
Bellazzi et al. [25]	HbA1c levels after 6 months of the control group decreased from 8.86 ± 2.15% to 7.95 ± 1,88% (p<0.05), while the HbA1c of intervention patients decreased from 8.31 ± 1.80% to 7.59 ± 1.46% (p<0.05). Although the difference in the mean HbA1c between groups is not statistically significant, different variances were observed after 6 months, with that of the intervention patients being lower than that of the control group (p<0.05).
Biermann et al. [26]	GHbA1c levels improved in both groups to 6.9 ± 1.3% after 4 months and 7.1 ± 0.7% after 8 months. Differences between the groups were not statistically significant.
Bergenstal et al. [27]	There were similar improvements in HbA1c levels between groups (p=0.18).
Bujnowska et al. [28]	There was no significant difference in HbA1c between groups (no p value reported). The patients' quality of life slightly improved in the intervention group and the control group, but there was no significant

	difference between them (no p value reported).
Ladyzynski and Wojcicki [29]	The HbA1c (p=0.72), MBG (p=0.46), and J indices (p=.51) were very similar in both groups.
**Non randomized studies**	
Bellazzi et al. [30]	The HbA1c and the insulin requirements were reduced in some of the subjects participating to the pilot study evaluation.
Liesenfeld et al. [31]	HbA1c dropped by 0.4% (p<0.05) at the end of the study. This corresponds to the reduction of MBG by 11 mg/dL even though the rate of hypoglycemic events was significantly reduced.
Meneghini et al. [32]	Prevalence of diabetic-related crises decreased approximately threefold (p<0.05) with a concomitant statistically significant decrease in HbA1c of 0.8% at 6 months (p=0.024) and 0.9% at 12 months (p=0.04).

**ASTHMA**	
**Large RCT with support**	
Rasmussen et al. [33]	The treatment and monitoring with the Internet-based management tool lead to significantly better improvement in the intervention group than in the two control groups regarding asthma symptoms (internet vs. specialist monitoring, p=0.02; internet vs. GP monitoring, p<0.001), quality of life (internet vs. specialist monitoring, p=0.03; internet vs. GP monitoring, p=0.04), lung function (internet vs. specialist monitoring, p=0.002; internet vs. GP monitoring, p<0.001), and airway responsiveness (internet vs. GP monitoring, p=0.02).
**Small RCTs with support**	
Jan et al. [34]	At the end of the trial, the intervention group decreased nighttime (p=0.028) and daytime symptoms (p=0.009); improved morning (p=0.017) and night PEF (p=0.01); increased adherence rates (p<0.05) and improved quality of life (p<0.05) when compared with patients in the control group.
Guendelman et al. [35]	The odds of having any limitation in activity during the 90-day trial were significantly (p=0.03) lower for children assigned to the intervention group. The intervention patients also were significantly (p=0.01) less likely to report peak flow readings in the yellow or red zone or to make urgent calls to the hospital (p=0.05).
Chan et al. [36]	Virtual group patients achieved excellent asthma therapeutic and disease control outcomes. Compared with those who received traditional office-based care, they were more adherent to diary submission

	(p<0.01) and had better inhaler scores at 52 weeks (p<0.01).
Ostojic et al. [37]	Mean FEV1 was similar in the 2 groups both before and after the study. However, control patients had significantly higher scores for cough (p<0.05) and night symptoms (p<0.05).
**Small RCT with no support**	
Chan et al. [38]	Peak flow values as a percentage of personal best values increased significantly from 84.3% for both groups over the first three months to 91.8% over the second three months (p<0.05). There was no change in quality of life reported by patients in both groups.
Willems et al. [39]	The telemonitoring program did not significantly decrease asthma symptoms or medical consumption, or improve asthma-specific quality of life.
**Non randomized study**	
Bruderman and Abboud [60]	In 19 patients (49%), analysis of the spirometric data detected early signs of asthmatic deterioration. Analysis of the spirometric data correlated with decisions to dispatch the mobile intensive care unit in 22 of 39 patients (56%).

**HEART FAILURE**	
**Large RCTs with support**	
Goldberg et al. [40]	Over the course of the 6-month follow-up, there were 26 deaths (18%) in the control group and 11 deaths (8%) in the intervention group, representing a 56% difference in mortality (p<0.003).
Cleland et al. [42]	Patients randomly assigned to receive usual care had higher one-year mortality (45%) than patients assigned to receive home telemonitoring (29%) (p=0.032).
Dansky et al. [44]	Results showed a greater reduction in diet-related symptoms (p=0.043), physical activity status symptoms (p=0.043), and medication effectiveness symptoms (p=0.001) for patients using telehomecare compared with control patients. However, no significant difference was observed with regard to mortality rates between the two groups (p=.11).
**Large RCT with no support**	
Benatar et al. [41]	Quality of life was significantly improved for both groups (p<0.01)
Soran et al. [43]	Mortality rate was not found to be significantly different between the control and intervention groups (p=.11).
**Small RCTs with support**	
Barnason et al. [61]	Intervention group participants had significantly higher adjusted mean self-efficacy scores (p<0.05) and

	adjusted mean levels of functioning ($p<0.05$) compared with the control group participants. Significantly higher exercise adherence ($p<0.01$) and lower reported stress ($p<0.01$) at 3 months after surgery was reported by intervention group subjects.
Woodend et al. [62]	After 3 months of monitoring, patients in the intervention group reported a higher level of quality of life than subjects in the control group ($p=0.03$).
Woodend et al. [63]	Patients in both the intervention and control groups showed significant improvements in overall LiHFe scores in the year after hospital discharge ($p<0.001$). Patients in the intervention group had significantly better functional status on both the overall score ($p=0.003$) and the physical subscale ($p=0.001$) of the LiHFe measure at 3 months than patients receiving usual care.
de Lusignan et al. [64]	Nine out of 10 patients in the intervention group showed a reduction in the SBP and 7 out of 10 showed a decrease in weight over the first 60 days of telemonitoring. There was no significant change in DBP or weight for patients in the control group. The quality of life of the telemonitoring group appeared to improve significantly while the control group deteriorated. No p values are reported in this study.
**Small RCTs with partial support**	
Scalvini et al. [47]	A significant reduction in instabilisations (55% vs. 24%, $p<0.0001$) was observed in intervention group compared with control group. However, no significant difference was found on mortality between groups.
Antonicelli et al. [48]	In the intervention group there was at follow-up a tendency towards an increase in left ventricular ejection fraction, accompanied by a reduction in DBP and heart rate ($p<0.01$). Patients in the intervention group displayed a tendency toward a lower death rate similar to that of subjects in the control group (p=ns). However, patients in intervention group were characterized at follow-up by a significantly better reported quality of life score as compared to baseline. This improvement was significantly greater as compared to control group patients ($p=0.046$).
**Small RCTs with no support**	
de Lusignan et al. [45]	There were no significant difference in the quality of life between the telemonitoring group and the control group after 1 year follow-up.
Schwarz et al. [46]	Patients were interviewed soon after discharge and 3 months later about effects of telemonitoring and depressive symptoms, quality of life, and caregiver mastery. There were no significant difference due to telemonitoring for any outcomes.
**Non randomized studies**	
Schofield et al. [65]	After enrollment, significant improvements were found in BP ($p<0.05$), weight ($p<0.01$), and shortness of

	breath rating (p=0.02).
Bondmass et al. [66]	At 6 and 12 months post enrollment, quality of life continued to remain significantly improved 26% (p<0.002) and 23% (p<0.003) from baseline, respectively.
Roth et al. [67]	Although only 38/118 patients were hospitalized, most patients reported a significant subjective improvement in their quality of life (no p value reported).
Scherr et al. [68]	At the end of the study, the patients' conditions were stable or improved when compared with baseline (no p value reported).

**HYPERTENSION**	
**Large RCTs with support**	
Friedman et al. [49]	No significant differences in changes of SBP but significantly higher decrease in DBP among intervention group (5.2 mmHg) vs. control group (0.8 mmHg) (p=0.02); non-adherent patients with medications were mostly affected. Among intervention group, decrease in SBP and DBP was significantly higher when medications adherence increased (12.7 and 5.5 mmHg) vs. decreased (5.5 and 0.6 mmHg).
Artinian et al. [50]	The intervention group had a greater reduction in SBP than the control group (p=0.04) from baseline to the 12-month follow-up. Although the intervention group had a greater reduction in DBP compared with the control group, the difference was not statistically significant (p=0.12)
Madsen et al. [51,52]	Patients in the intervention group had higher mean scores in the bodily pain domain (SF-36 questionnaire) than patients in the control group, indicating less pain and interference with activities among telemonitored patients (p=0.026). In both groups, systolic BP decreased significantly from baseline to follow-up. The decrease was -11.9 mmHg in the intervention group and -9.6 mmHg in the control group (mean difference -2.3, p=0.225). As a result, the authors conclude that antihypertensive treatment based on telemonitoring of home BP was as effective as usual monitoring of office BP with regards to reduction of BP (2008b).
**Small RCTs with support**	
Rogers et al. [53]	Significant difference in mean arterial BP: decrease by 2.8 mmHg and increase by 1.3 mmHg for telemonitoring and usual care patients, respectively. Significant differences in mean DBP and SBP: decrease by 2 mmHg (intervention) and increase by 2.1 mmHg (control) for DBP, and decrease by 4.9 mmHg (intervention) and 0.1 mmHg (control) for SBP. Intervention patients were 2.32 times more likely to have improvement in DBP and 2.52 times in SBP. Greater significant effects on mean arterial BP

	among African American. Frequent significant changes in medications; 40% (intervention) vs. 28% (control) received change in medication type. 33% (intervention) vs. 7% (control) received changes in dosage.
Artinian et al. [54]	Greater clinical and statistical improvement in BP (significant drop in SBP and DBP) among the telemonitoring and community monitoring groups compared to usual care over 3 months. Statistically significant decrease in SBP and DBP by 24.7 mmHg and 14.62 mmHg, respectively, in the telemonitoring group as compared to the two other groups.
**Non randomized studies**	
Naef et al. [69]	Arterial blood pressure and pulse rates measured at home significantly lower than those measured in the clinic ($p<0.05$). Consistent results obtained by patients. No unexpected fatal outcomes and maternal complications. Transmitted blood pressure values were used to adjust medications.
Port et al. [70]	Telemonitoring allowed identifying differences in compliance with treatment. In terms of long term treatment (drug) effectiveness, 16 patients (32%) had weekly decrease in SBP vs. 21 patients (42%) with no decrease and 13 patients (26%) with an increase. Treatment most successful among most compliant patients who had largest decrease in SBP (3.4 mmHg) and DBP (2.8 mmHg) with drug intake.
Nakamoto et al. [71]	SBP and DBP measured at home were significantly lower than BP measured in the clinic. A strong correlation was observed between BP measured at home and in the outpatient clinic. BP home telemonitoring allowed detection of "white coat" effect among 43% of patients.
Bondmass et al. [72]	Reported clinical effectiveness. Significant decrease in SBP from 154.1 mmHg at baseline to 144.8, 142.4, and 141.4 mmHg at 30, 60, and 90 days. Significant decrease in DBP from 89.9 mmHg at baseline to 85.3, 83.9, and 83.2 mmHg at 30, 60, and 90 days. SBP control was maintained by 60%, 53.3%, and 50% of patients at 30, 60, and 90 days. DBP control was maintained by 70%, 63%, and 67% of patients at 30, 60, and 90 days. No detrimental changes in heart rates were observed. A total of 1099 alarms were received due to abnormal readings (above or below limits), out of which 61% were before 8:00 AM & / or after 5:00PM. 42 medication changes took place. No significant change in quality of life ranked as satisfying.
Port et al. [73]	53% of patients showed a decrease in BP as compared to 35% who had increase in BP and 12% with no consistent trend.
Mengden et al. [74]	In contrast to office BP, telemonitoring of BP allowed the early identification of responders (e.g., the majority of responders could be identified after 2-3 weeks). BP reduction was greater for office BP than

	for telemonitoring patients ($p<0.05$).
Dalton et al. [75]	On almost every occasion, the blood pressure measured at home was lower than that previously measured in the hospital clinic (no p value reported).
