# Supplementary material for: Clinical Effects of Home Telemonitoring in the Context of Diabetes, Asthma, Heart Failure and Hypertension: A Systematic Review
Source: J Med Internet Res. 2010 Jun 16;12(2):e21. doi: 10.2196/jmir.1357 (PMC2956232; doi:10.2196/jmir.1357)
Supplement: Supplementary file 2 [file jmir_v12i2e21_app2.pdf]

Multimedia Appendix 1: List of excluded studies

Other forms of telehomecare intervention (ex. telephone-based intervention or call center, teleconsultation or virtual visit; tele-education, physical monitoring, etc.)

1. Young R, et al. Pro-active call center treatment support (PACCTS) to improve glucose control type 2 diabetes: a randomized controlled trial. Diabetes Care 2005;28:278-282.
2. Orlov OI, et al. Wireless ECG monitoring by telephone. Telemedicine Journal and e-Health 2001;7:33-38.
3. Finkelstein SM, et al. Telehomecare: Quality, Perception, Satisfaction. Telemedicine Journal and e-Health 2004;10(2):122-128.
4. Roglieri JL, et al. Disease management interventions to improve outcomes in congestive heart failure. The American Journal of Managed Care 1997;3:1831-9.
5. Izquierdo RE, et al. A comparison of diabetes education administered through telemedicine versus in person. Diabetes Care 2003;26(4):1002-7.
6. LaFramboise LM, et al. Comparison of Health Buddy with traditional approaches to heart failure management. Family and Community Health 2003;26(4):275-88.
7. Jenkins RI, et al. Assessing elderly patients with congestive heart failure via in-home interactive telecommunication. Journal of Gerontological Nursing 2001;(January):21-27.
8. Dansky KH, Bowles KH. Lessons learned from a telehomecare project. CARING Magazine 2002;(April):18-22.
9. Bowles KH, Dansky KH. Teaching self-management of diabetes via telehomecare. Home Healthcare Nurse 2002;20(1):36-42.
10. Finkelstein SM, et al. Telehomecare: connecting the home and the home care agency. CARING Magazine 2000;(July):32-5.
11. Demiris G. et al. Change of patients' perceptions of Telehomecare. Telemedicine Journal and e-Health 2001;7(3):241-8.
12. Whitten P. et al. Home telenursing in Kansas: patients' perceptions of uses and benefits. Journal of Telemedicine and Telecare 1997;3(Suppl.1):67-9.
13. Johnson-Mekota JL, et al. A nursing application of telecommunications: measurement of satisfaction for patients and providers. Journal of Gerontological Nursing 2001;(January):28-33.
14. Young NL, et al. Telehomecare: a comparison of three Canadian models. Telemedicine Journal and e-Health 2004;10(1):45-52.
15. Demiris G. et al. The nature of communication in virtual home care visits. Proceedings of the 2001 AMIA Conference, 135-138.
16. Kormowski R, et al. Intensive home-care surveillance prevents hospitalization and improves morbidity rates among elderly patients with severe congestive heart failure. American Heart Journal 1996;129(4):762-6.
17. Bartholomew LK, et al. Watch, Discover, Think, and Act: evaluation of computer-assisted instruction to improve asthma self-management in inner-city children. Patient Education and Counseling 2000;39:269-80.
18. Meigs JB, et al. A controlled trial of web-based diabetes disease management: the MGH diabetes primary care improvement project. Diabetes Care 2003;26(3):750-7.
19. Arnaert A, Delesie L. Effectiveness of Video-Telephone Nursing Care for the Homebound Elderly. CNJR 2007;39(1):20-36.

20. Ewald S, et al. Relationship between the frequency of blood pressure self-measurement and blood pressure reduction with antihypertensive therapy. Clin Drug Invest 2006;26(8):439-46.
21. Hebert MA, et al. Successes and challenges in a field-based, multi-method study of home telehealth. Journal of Telemedicine and Telecare 2004;10(Suppl.1):41-4.
22. Young L, et al. Post-surgical telehealth support for children and family care-givers. Journal of Telemedicine and Telecare 2007;13:15-9.
23. Vincent JA, et al. Diagnostic and cost effectiveness of telemonitoring the pediatric pacemaker patient. Pediatric Cardiology 1997;18:86-90.
24. Napoli A, et al. Blood pressure monitoring in diabetic pregnancy. Ann 1st Sper Sanita 1997;33(3):337-341.
25. Pickering TG, et al. Telephone-linked home blood pressure monitoring may improve management. AJH 1999; 12(4, Part 2):163A.
26. Johnston B, et al. Outcomes of the Kaiser Permanente Tele-Home health research project. Arch Fam Med 2000;9(January):40-5.
27. Riegel B, et al. Effect of a standardized nurse case-management telephone intervention on resource use in patients with chronic heart failure. Arch Intern Med 2002;162(March 25):705-12.
28. GESICA Investigators. Randomised trial of telephone intervention in chronic heart failure: DIAL trial. BMJ 2007; doi:10.1136/bmj.38516.398067.E0, 5 pages.
29. Dunagan C, et al. Randomized trial of a nurse-administered, telephone-based disease management program for patients with heart failure. Journal of Cardiac Failure 2005;11(5):358-65.
30. Riegel B, et al. Randomized controlled trial of telephone case management in Hispanics of Mexican origin with heart failure. Journal of Cardiac Failure 2006;12(3):211-9.
31. Duffy JR, et al. Research challenges and lessons learned from a heart failure telehomecare study. Home Healthcare Nurse 2008;26(1):58-65.
32. Oudshoorn N. Diagnosis at a distance: the invisible work of patients and healthcare professionals in cardiac telemonitoring technology. Sociology of Health & Illness 2008;30(2):272-88.
33. Vincent C, et al. Public telesurveillance service for frail elderly living at home, outcomes and cost evolution: a quasi-experimental design with two follow-ups. Health and Quality of Life Outcomes 2006;4:41. doi: 10.1186/1477-7525-4-41.
34. Nakamura K, et al. The effectiveness of videophones in home healthcare for the elderly. Medical Care 1999;37(2):117-25.
35. DeBusk RF, et al. Care Management for low-risk patients with heart failure. Annals of Internal Medicine 2004;141(8):606-13.
36. Terschüren C, et al. Implementing telemonitoring in the daily routine of a GP practice in a rural setting in northern Germany. Journal of Telemedicine and Telecare 2007;13(4):197-201.
37. Caouette A, et al. Use of telemonitoring by elders at home: actual practice and potential. Can J Occup Ther 2007;74(5):383-92.
38. Ladyzynski P, et al. Mobile telecare system for intensive insulin treatment and patient education. First applications for newly diagnosed type 1 diabetic patients. Int J Artif Organs 2006;29(11):1074-81.
39. Zarter P, et al. Performance of an autonomous telemonitoring system in children and young adults with congenital heart diseases. Pacing Clin Electrophysiol 2008;31(10):1291-9.

40. Abraham C, Rosenthal DA. Merging home and health via contemporary care delivery: program management insights on a home telehealth project. Comput Inform Nurs 2008;26(5):273-81.
41. Casey FA. Home support for children with complex congenital heart disease using videoconferencing via broadband: initial results. Journal of Telemedicine and Telecare 2008;14(3):140-2.

No telemonitoring intervention or no effects presented / focus on the technology being implemented:

42. Barbaro, V, et al. A portable unit for remote monitoring of pacemaker patients. Journal of Telemedicine and Telecare 1997;3:96-102.
43. Vouyioukas D. et al. Pervasive e-health services using the DVB-RCS communication technology. Journal of Medical Systems 2007;31(4):237-46.
44. Johnson P, Andrews DC. Remote continuous physiological monitoring in the home. Journal of Telemedicine and Telecare 1996;2:107-13.
45. Farmer A, et al. A real-time, mobile phone-based telemedicine system to support young adults with type 1 diabetes. Informatics in Primary Care 2005;13:171-7.
46. Ishikawa K, et al. An experimental study on home health care support information system construction. MEDINFO 1995 Proceedings, 581-5.
47. Chaudhry SI, et al. Randomized trial of telemonitoring to improve heart failure outcomes (Tele-HF): study design. Journal of Cardiac Failure 2007;13(9):709-14.
48. Canady LM. Implementing a home telemonitoring program. Home Healthcare Nurse 2008;26(4):231-6.
49. Subramanian U, et al. Research in home-care telemedicine: challenges in patient recruitment. Telemedicine Journal and e-Health 2004;10(2):155-61.
50. Shuicai W, et al. An internet-based telemonitoring system of multiphysiological parameters. Telemedicine Journal and e-health 2007;13(4):451-9.
51. Barlow J, et al. A systematic review of the benefits of home telecare for frail elderly people and those with long-term conditions. Journal of Telemedicine and Telecare 2007;13:172-9.
52. Chung WY, et al. A cell phone based health monitoring system with self analysis processor using wireless sensor network technology. Conf Proc IEEE Eng Med Biol Soc 2007;2705-8.
53. Dalton AF, et al. A clinical evaluation of a remote mobility monitoring system based on SMS messaging. Conf Proc IEEE Eng Med Biol Soc 2007;2327-30.
54. Fursse J, et al. Early experience in using telemonitoring for the management of chronic disease in primary care. Journal of Telemedicine and Telecare 2008;14(3):122-4.
55. Almudevar A, et al. Home monitoring using wearable radio frequency transmitters. Artif Intell Med 2008;42(2):109-20.

Patients with multiple illnesses:

56. Kobb R, Enhancing elder chronic care through technology and care coordination: report from a pilot. Telemedicine Journal and e-health 2003;9(2):189-95.
57. Zhang Y, et al. First trial of home ECG and blood pressure telemonitoring system in Macau. Telemedicine Journal and e-health 1997;3(1): 67-72.

58. Fursse J, et al. Early experience in using telemonitoring for the management of chronic disease in primary care. Journal of Telemedicine and Telecare 2008;14(3):122-4.
59. Chumbler NR, et al. The association of home-telehealth use and care coordination with improvement of functional and cognitive functioning in frail elderly men. Telemed J E Health 2004;10(2):129-37.
60. Noel HC, et al. Home telehealth reduces healthcare costs. Telemed J E Health 2004;10(2):170-83.
61. Finkelstein SM, et al. Home telehealth improves clinical outcomes at lower cost for home healthcare. Telemed J E Health 2006;12(2):128-36.
62. Trudel M, et al. A mobile phone based remote patient monitoring system for chronic disease management. Medinfo 2007;12(Pt 1):167-71.
63. Stricklin MLV, et al. Home Talk/Healthy Talk: improving patients' health status with telephone technology. Home Healthcare Nurse 2000;18(1):53-61.
64. Finkelstein SM, et al. Home telehealth improves clinical outcomes at lower cost for home healthcare. Telemed J E Health 2006;12(2):128-36.

Editorials or essays:

65. Field MJ, Grigsby J. Telemedicine and remote patient monitoring. JAMA 2002;288(4):423-5.
66. Bowles KH, Baugh AC. Applying research evidence to optimize telehomecare. Journal of Cardiovascular Nursing 2007;22(1):5-15.
67. Meystre S. The current state of telemonitoring: a comment on the literature. Telemedicine Journal and e-health 2005;11(1):63-9.
68. Helms TM, et al. Telemonitoring of cardiovascular diseases in Germany. Herz 2007;32(8):641-9.
